# The Polarization Properties of the Reflection Spectra of Single-Layer MoS_2_ and ReS_2_ on SiO_2_/Si and Quartz Substrates

**DOI:** 10.1186/s11671-020-3280-8

**Published:** 2020-02-17

**Authors:** Yafang Shi, Longlong Wang, Xiaofen Qiao, Shuai Li, Yi Liu, Xiaoli Li, Xiaohui Zhao

**Affiliations:** 1 National-Local Joint Engineering Laboratory of New Energy Photoelectric Devices, College of Physics Science & Technology, Hebei University, Baoding, 071002 People’s Republic of China; 2 State Key Laboratory of Superlattices and Microstructures, Institute of Semiconductors, Chinese Academy of Sciences, Beijing, 100083 People’s Republic of China

**Keywords:** MoS_2_, ReS_2_, Reflection spectra, Polarization angle, Anisotropic material, Isotropic material

## Abstract

MoS_2_ and ReS_2_ are typical transition metal chalcogenides with many excellent electrical and optical properties. Due to different lattice symmetries, ReS_2_ offers one more dimension than MoS_2_ to tune its physical properties. In this paper, we studied the polarized reflection spectra in single-layer MoS_2_ and ReS_2_. The explicit difference identifies strong angle-dependent properties in single-layer ReS_2_ distinct from single-layer MoS_2_. The results of samples on both SiO_2_/Si substrate and quartz substrate show single-layer ReS_2_ is in-plane anisotropic and the change period of reflection intensity is estimated with the polarization angles.

## Introduction

The rapid progress of graphene research has stimulated interest in other several types of two-dimensional layered materials. Recently, transition metal dichalcogenides (TMDs) have attracted considerable attention since the observation of remarkable electronic and optical properties [1–3]. These TMD crystals can be grown or mechanically exfoliated to monolayer thickness, similar to the exfoliation of graphene. However, in contrast to graphene, monolayer TMDs consist of more than one element, which makes their physical properties more complex than graphene. Among the TMDs, MoS_2_ is the most extensively studied, where one Mo plane is sandwiched between two S planes usually with a 2H-structure [4]. In contrast to these high-symmetry hexagonal structures such as MoS_2_, another kind of TMDs such as ReS_2_ is attracting much interest, which exhibits a distorted 1T’-structure [5]. The upper and lower S atoms sandwich the middle layer of Re atoms with a hexagonal structure having an additional Peierls twist [5]. This is because the rhenium atom possesses one extra valence electron, leading to the formation of additional Re–Re bonds in ReS_2_ (atomic structure diagram of a single-layer MoS_2_ and ReS_2_ is shown in Additional file 1: Figure S1.) The reduced symmetry in ReS_2_ induces significant in-plane anisotropy and therefore adds an additional degree of freedom, which makes ReS_2_ an interesting material for the fabrication of FETs and polarization-sensitive photodetectors [5, 6]. In this paper, we probed the polarization properties of single-layer (abbreviated as the notation SL) MoS_2_ and ReS_2_ flakes by angular-dependent reflection spectra measurements on SiO_2_/Si and quartz substrates. Our results will shed light on the new effects in those strongly anisotropic layered materials and can empirically be used to identify the crystal orientation.

## Materials and Methods

The MoS_2_ and ReS_2_ flakes with different numbers of layers in this paper were exfoliated from bulk MoS_2_ and ReS_2_ crystals by micromechanical cleavage method and were prepared on substrates. The interaction between samples and substrates was different and the influence of substrates on experimental results should be considered. Thus, we selected two kinds of substrates: one is the Si {100} substrate covered with an 89 nm SiO_2_ and the other is the quartz crystal with a thickness of 1 mm, to support MoS_2_ and ReS_2_ flakes (the optical microscopic images of SL MoS_2_ and SL ReS_2_ flakes supported on SiO_2_/Si substrate and supported on quartz substrate are shown in Additional file 1: Figure S2.) The SL dichalcogenides have a thickness between 0.6 and 0.7 nm which are extremely sensitive to measurement accuracy of measuring instruments. We used ultra-low frequency Raman spectroscopy [7, 8] (the ultra-low frequency Raman spectra of SL MoS_2_ and SL ReS_2_ flakes supported on SiO_2_/Si substrate and supported on quartz substrate are shown in Additional file 1: Figure S3.) and photoluminescence (PL) spectroscopy [8, 9] (the PL spectra of SL MoS_2_ and SL ReS_2_ flakes supported on SiO_2_/Si substrate and supported on quartz substrate are shown in Additional file 1: Figure S4.) to accurately determine the SL MoS_2_ and ReS_2_ flakes.

Reflection spectrum measurements were performed in a backscattering geometry using a Jobin-Yvon HR800 micro-Raman system. The tungsten halogen lamp was used as a light source with the spot size below 2 μm. The objective of × 100 (NA = 0.9) was used to ensure the accuracy of tests with the size of samples above 5 μm. The best reflected light signal was achieved by focusing the microscope to get maximum peak intensity. The reflection spectra were measured from the samples and bare substrates in the broad wavelength range of 400–800 nm. The 600 lines per millimeter grating was used, which enables one to have each CCD pixel to cover 1 nm. A polarizer was placed on the light path in front of the sample. By continuously rotating the polarizer from 0 to 360°, polarization directions of incident and reflected light were simultaneously varied with polarization angles from 0 to 360°. When the polarizer was rotated to an angle, the reflection spectra of the sample (SL MoS_2_ or SL ReS_2_) and the substrate (SiO_2_/Si or quartz) were measured once. All of the polarization reflection spectra were measured under the condition of keeping the lamp intensity unchanged. We used *R* (sam + sub) and *R* (sub) to respectively indicate the reflection intensities of samples and bare substrates and used the optical contrast method to normalize the data by the formula of *R*_OC_ = 1 − *R* (sam + sub)/*R* (sub) (the substrate is SiO_2_/Si) or *R*_OC_ = *R* (sam + sub)/*R* (sub) − 1 (the substrate is quartz). In the following studies, the angular-dependent optical contrasts of SL MoS_2_ and ReS_2_ on different substrates were demonstrated respectively.

## Results and Discussion

### SL MoS_2_ on SiO_2_/Si Substrate

**Fig. 1:**
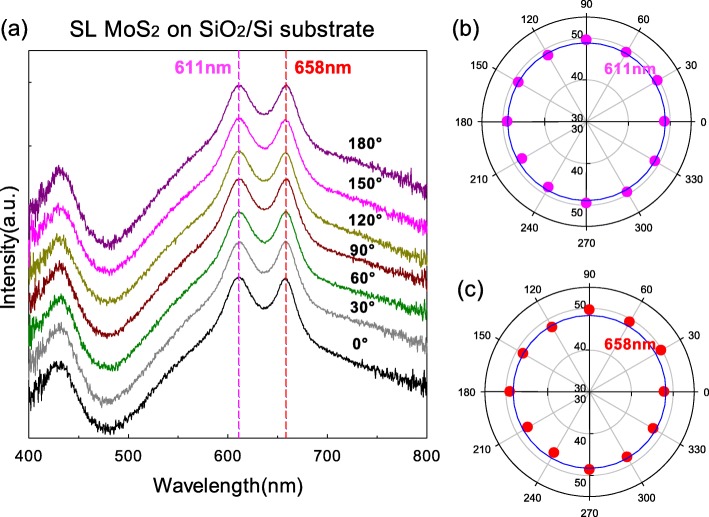
**a** The polarization optical contrast curves of SL MoS_2_ flakes supported on SiO_2_/Si substrate. **b** The intensity variation at ~ 611 nm from 0 to 360°. **c** The intensity variation at ~ 658 nm from 0 to 360°

We firstly measured the polarization reflection spectra of SL MoS_2_ supported on SiO_2_/Si substrate by continuously rotating the polarizer from 0 to 360°. The polarizer was rotated once every 30°. Figure 1 a shows the variation of optical contrasts with polarization angles from 0 to 180°. The original curves were overlapping each other and the processed curves were offset for clarity. There are two peaks at ~ 611 nm and ~ 658 nm due to A and B exciton emission [10, 11]. We selected them as references and showed their intensities with the polarization angles from 0 to 360° in Fig. 1 b and c by pink and red circles, respectively. The intensities of two peaks are basically unchanged, which is we should predict since the SL MoS_2_ is hexagonally symmetrical.

### SL ReS_2_ on SiO_2_/Si Substrate

**Fig. 2:**
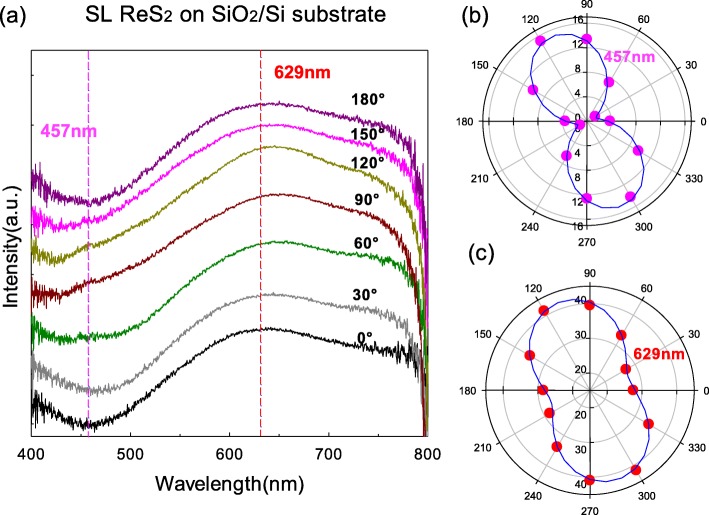
**a** The polarization optical contrast curves of SL ReS_2_ flakes supported on SiO_2_/Si substrate. **b** The intensity variation at ~ 457 nm from 0 to 360°. **c** The intensity variation at ~ 629 nm from 0 to 360°

The polarization reflection spectra of SL ReS_2_ supported on SiO_2_/Si substrate were measured as followed. The optical contrast curves of SL ReS_2_ flake with varying polarization angles from 0 to 180° are shown in Fig. 2 a and are offset for clarity. There is a valley at ~ 457 nm and a peak at ~ 629 nm [12] suggesting that SL ReS_2_ crystallizes in a different crystal structure from SL MoS_2_. The intensities at ~ 457 nm and ~ 629 nm changed as the polarization angle changed. Taking them as references, we showed their intensities with the polarization angles from 0 to 360° in Fig. 2 b and c by pink and red circles, respectively. Both of the intensities at two positions show polarization dependence on the polarization angles, which is directly resulted from the low crystal symmetry in SL ReS_2_. The in-plane distortion of SL ReS_2_ lattice is expected to affect profoundly the interlayer coupling in multilayer ReS_2_ crystals because the similar polarization dependence has been found in the optical contrast curves of anisotropic-like-stacked 2 L ReS_2_ flakes supported on SiO_2_/Si substrate [12] and even in the ultra-low frequency Raman spectra and PL spectra of isotropic-like-stacked 2 L ReS_2_ flakes [8].

We fitted the function of the intensities at ~ 457 nm and ~ 629 nm as the polarization angles by a first-order Fourier formula: *f*(*θ*) = a0 + a1 × cos(*θ* × *w*) + b1 × sin(*θ* × *w*), where *θ* is the polarization angle; a0, a1, and b1 are the amplitudes; and *w* is the frequency. The positions of minimum and maximum intensities were read as 20° and 110°, respectively, at both of ~ 457 nm and ~ 629 nm. The fitted curves were also plotted in Fig. 2 b and c with blue lines. At ~ 457 nm, a0 = 8.269, a1 = − 4.878, b1 = − 4.585, and *w* = 0.0348, and at ~ 629 nm, a0 = 34.27, a1 = − 5.99, b1 = − 4.747, and *w* = 0.03525. They have the basically identical change period with the polarization angles due to the nearly equal *w*. It should be derived from the distorted structure in the SL ReS_2_.

### SL MoS_2_ on Quartz Substrate

Because SiO_2_/Si substrate is opaque, the incident light passed through interfaces of air/sample and sample/substrate and finally was absorbed by the substrate. Meanwhile, the reflected light was collected from each interface and finally transmitted into the air. The optical interference occurred in the multilayered structures and physical properties of the substrate were included in the outgoing-reflected signals in addition to the sample [12]. The SiO_2_/Si substrate was a polarized substrate although we used the optical contrast method to normalize the data by the formula of *R*_OC_ = 1 − *R* (sam + sub)/*R* (sub). In order to eliminate the disturbance of polarized properties from the substrate, we then measured the polarization reflection spectra of SL MoS_2_ and ReS_2_ on the quartz substrate due to the transparency and isotropy of the quartz substrate.

**Fig. 3:**
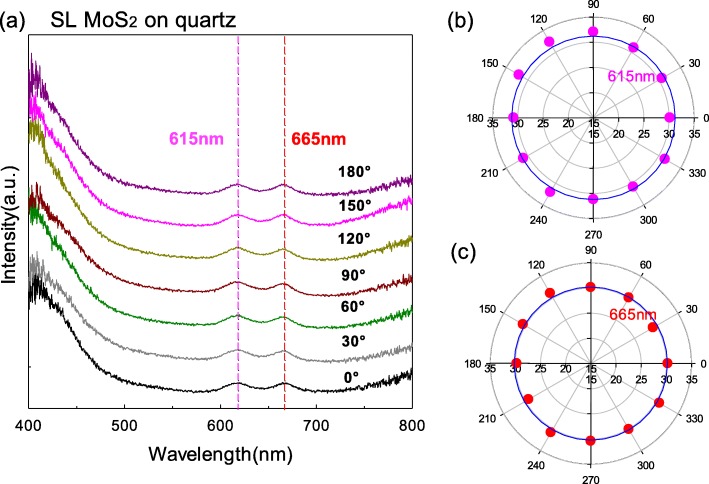
**a** The polarization optical contrast curves of SL MoS_2_ flakes supported on quartz substrate. **b** The intensity variation at ~ 615 nm from 0 to 360°. **c** The intensity variation at ~ 665 nm from 0 to 360°

Since the quartz substrate is transparent, the sample stage should be placed suspended to ensure transparency during measuring. The incident light passed through interfaces of air/sample, sample/substrate, and substrate/air and finally was absorbed by the air to avoid disturbing the collecting of reflected light. We used the formula of *R*_OC_ = *R* (sam + sub)/*R* (sub) − 1 to normalize the data. Figure 3 a shows the polarized optical contrast curves of SL MoS_2_ flake on the quartz substrate with varying polarization angles from 0 to 180°. As can be seen, there are also two peaks related to A and B exciton at ~ 615 nm and ~ 665 nm, respectively. Their position has some shift towards long wavelength than that supported on SiO_2_/Si substrate due to interference effects on different substrates [11]. We plotted their intensities with the polarization angles in Fig. 3 b and c. The intensities of two peaks are almost no change as the polarization angle changes, which indicates that in-plane isotropic properties of SL MoS_2_ are unchangeable when they are attached to whatever substrates.

### SL ReS_2_ on Quartz Substrate

**Fig. 4:**
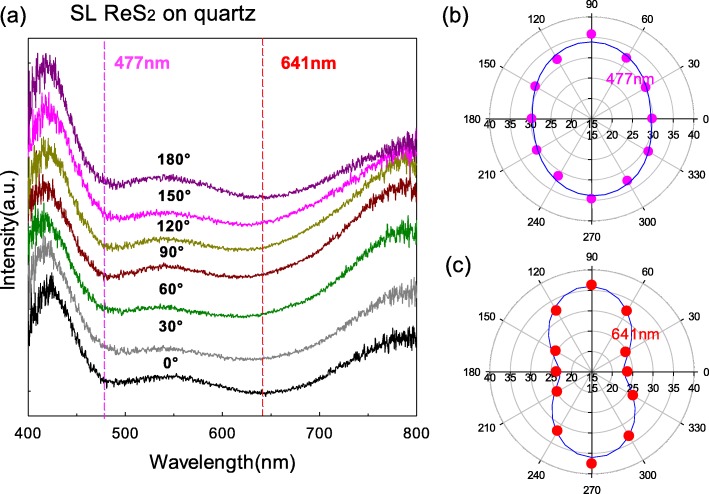
**a** The polarization optical contrast curves of SL ReS_2_ flakes supported on quartz substrate. **b** The intensity variation at ~ 477 nm from 0 to 360°. **c** The intensity variation at ~ 641 nm from 0 to 360°

Figure 4 a shows the polarized optical contrast curves of SL ReS_2_ flake on the quartz substrate, in which there are two valleys at ~ 477 nm and ~ 641 nm, respectively. The difference of features between supported on the quartz substrate and supported on SiO_2_/Si substrate is also due to interference effects on different substrates [11]. Figure 4 b and c show the intensities of two valleys with the polarization angles. Both of them show polarization dependence on the polarization angles, which indicates that SL ReS_2_ is in-plane anisotropic regardless of substrates. We fitted the relation of the intensities at ~ 477 nm and ~ 641 nm with the polarization angles by a first-order Fourier formula: *f*(*θ*) = a0 + a1 × cos(*θ* × *w*) + b1 × sin(*θ* × *w*), where a0 = 0.3168, a1 = − 0.02215, b1 = − 0.0004139, and *w* = 0.03422 at ~ 477 nm and a0 = 0.2941, a1 = − 0.06608, b1 = − 0.005685, and *w* = 0.0349 at ~ 641 nm. The positions of minimum and maximum intensities were read as 0° and 90°, respectively, at both of ~ 477 nm and ~ 641 nm. The fitted curves were also plotted in Fig. 4 b and c with blue lines. The *w* is basically identical at both ~ 477 nm and ~ 641 nm and nearly equal to that at ~ 457 nm and ~ 629 nm of SL ReS_2_ flakes supported on SiO_2_/Si substrate, which means that the polarized properties in SL ReS_2_ flakes exhibit a change tendency in the sin or cos function as the polarization angle changes from 0 to 360° and the period is uniform when they are attached to whatever substrates.

## Conclusions

In conclusion, SL MoS_2_ and ReS_2_ on SiO_2_/Si substrate and on quartz substrate have been studied by polarization reflection spectra, which identify a significant in-plane isotropy in SL MoS_2_ due to a hexagonal structure and in-plane anisotropy in SL ReS_2_ due to an additional distorted structure with a hexagonal structure. According to the polarized optical contrast curves with the polarization angles, there are some wavelength-dependent peaks or valleys in SL MoS_2_ and ReS_2_ predicted by different crystal structures. The variation of intensities at peaks or valleys with the polarization angles confirms the existence of different angle-dependent properties in SL MoS_2_ and ReS_2_. The same properties exist in some SL 2D materials having a similar structure with MoS_2_ such as WS_2_, MoSe_2_, and WSe_2_, and having a similar structure with ReS_2_ such as ReSe_2_ and WTe_2_. There are many other SL 2D materials that have other types of asymmetric lattice structures, such as BP and SnSe, which have strongly buckled honeycomb sheets with “troughs” running along the *y*-axis. These samples might also show anisotropic features. It implies that some new polarization-dependent electronic devices may soon be realized and promoted considering the wide variety of samples.

## Supplementary information

**Additional file 1: Figure S1** Atomic structure diagram of SL MoS2 and SL ReS2. **Figure S2** The optical microscopic images of SL MoS2 and SL ReS2 flakes supported on SiO2/Si substrate and supported on quartz substrate. **Figure S3** The ultralow-frequency Raman spectra of SL MoS2 and SL ReS2 flakes supported on SiO2/Si substrate and supported on quartz substrate. **Figure S4** The PL spectra of SL MoS2 and SL ReS2 flakes supported on SiO2/Si substrate and supported on quartz substrate.

## Data Availability

The SL MoS_2_ and SL ReS_2_ flakes were exfoliated from bulk MoS_2_ and ReS_2_ crystals by micromechanical cleavage method, prepared on two kinds of substrates: the Si {100} substrate covered with a 89-nm SiO_2_ and the quartz crystal with a thickness of 1 mm, and identified by ultra-low frequency Raman spectroscopy and PL spectroscopy. Reflection spectra measurements were performed in a backscattering geometry using a Jobin-Yvon HR800 micro-Raman system. The tungsten halogen lamp was used as a light source. A polarizer was placed on the light path in front of the sample. By continuously rotating the polarizer from 0 to 360°, the polarization reflection spectra of samples and substrates were measured and the optical contrast method was used to normalize the data.

## Abbreviations

PL: Photoluminescence
SL: Single-layer
TMDs: Transition metal dichalcogenides
