# Supplementary material for: The Polarization Properties of the Reflection Spectra of Single-Layer MoS2 and ReS2 on SiO2/Si and Quartz Substrates
Source: Nanoscale Res Lett. 2020 Feb 17;15:43. doi: 10.1186/s11671-020-3280-8 (PMC7026318; doi:10.1186/s11671-020-3280-8)
Supplement: Supplementary file 1 — Additional file 1: Figure S1 Atomic structure diagram of SL MoS2 and SL ReS2. Figure S2 The optical microscopic images of SL MoS2 and SL ReS2 flakes supported on SiO2/Si substrate and supported on quartz substrate. Figure S3 The ultralow-frequency Raman spectra of SL MoS2 and SL ReS2 flakes supported on SiO2/Si substrate and supported on quartz substrate. Figure S4 The PL spectra of SL MoS2 and SL ReS2 flakes supported on SiO2/Si substrate and supported on quartz substrate. [file 11671_2020_3280_MOESM1_ESM.pdf]

Additional files

MoS2 is the most extensively studied, where one Mo plane is sandwiched between two S planes usually with a 2H-structure. In contrast to these high-symmetry hexagonal structures such as MoS_2, another kind of TMDs such as ReS_2 are attracting much interest, which exhibits a distorted 1T'-structure. The upper and lower S atoms sandwich the middle layer of Re atoms with a hexagonal structure having an additional Peierls twist. This is because the rhenium atom possesses one extra valence electron, leading to the formation of additional Re-Re bonds in ReS_2.

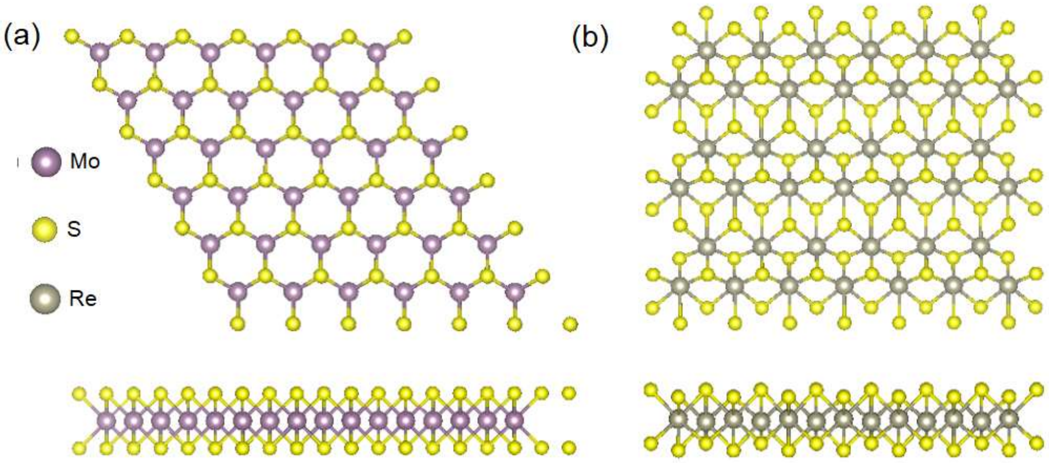

Figure f1. Atomic structure diagram of SL MoS_2 and SL ReS_2.

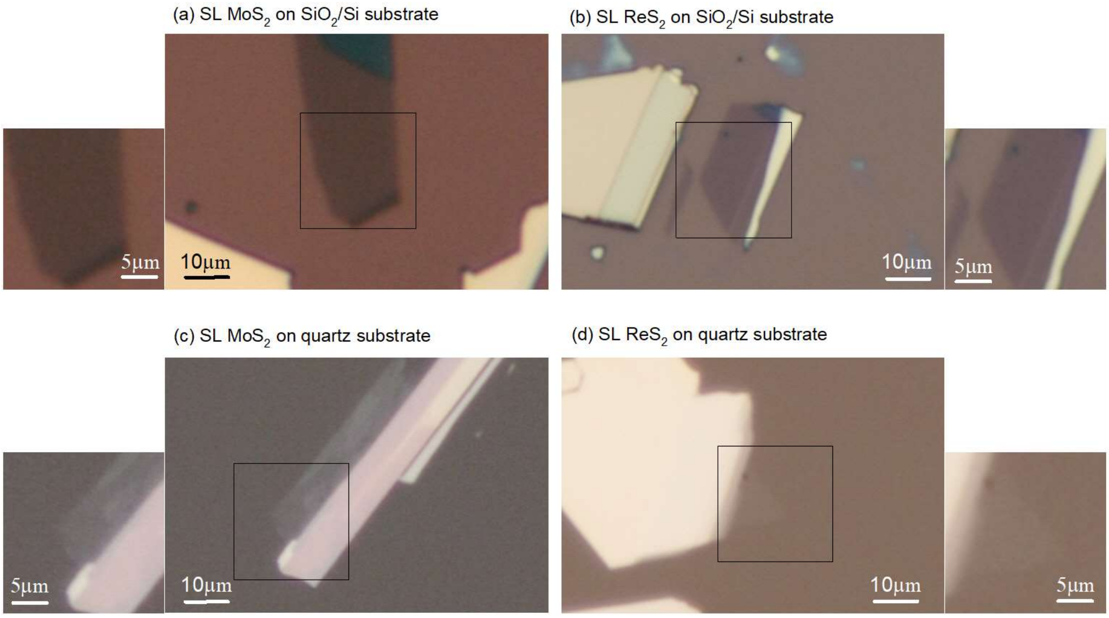

Figure f2. The optical microscopic images of SL MoS_2 and SL ReS_2 flakes supported on SiO_2/Si substrate and supported on quartz substrate.

The ultralow-frequency Raman technique can be used to determine accurately the layer number of MoS_2 and ReS_2 flakes. (See the papers as Adv. Funct. Mater. 2017, 1604468; Nanoscale, 2016, 8, 8324–8332; Phys. Rev. B 2013, 87, 115413.) Raman spectra of layered 2D materials (such as MoS_2 and ReS_2) consists of the high-frequency modes (above $100cm^{-1}$) and ultralow-frequency modes (below $60cm^{-1}$). The ultralow-frequency modes correspond to the relative motions of the planes themselves in N-layers (NL, N >1) MoS_2 and ReS_2 with several stacking orders, either parallel or perpendicular to the plane, such as the shear (S) modes and the LB modes. The S and LB vibrations are an intrinsic property of NL (N >1) MoS_2 and ReS_2 and are strongly dependent on N (N >1) in the peak position. In particular, the S and LB modes do not exist in SL MoS_2 and ReS_2. Our samples in this paper were pre-estimated by the ultralow-frequency Raman measurements.

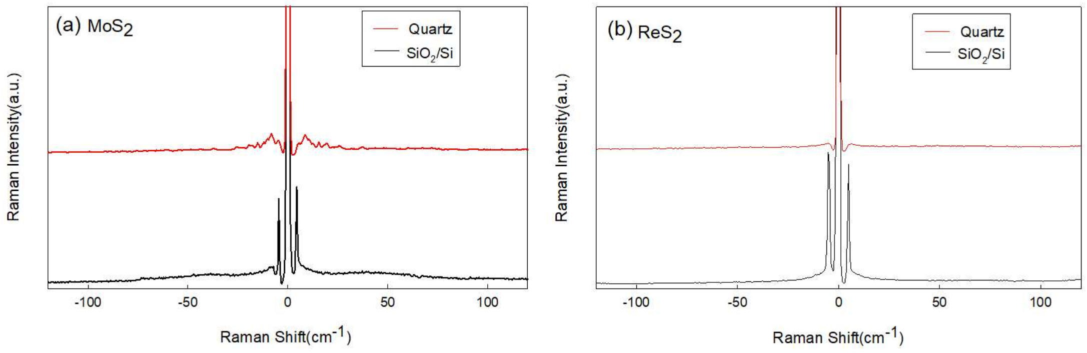

Figure f3. The ultralow-frequency Raman spectra of SL MoS_2 and SL ReS_2 flakes supported on SiO_2/Si substrate and supported on quartz substrate.

The PL spectra also can be used to determine the layer number of MoS_2 and ReS_2 flakes. (See the papers as Adv. Funct. Mater. 2017, 1604468; Nanoscale, 2016, 8, 8324–8332). MoS_2 are indirect gap semiconductors in bulk, but transform to direct gap semiconductors when thickness is reduced to monolayer. The PL spectrum of SL MoS_2 consists of a direct excited feature (A and B excitons) at higher energy but no indirect excited feature (I peak) at lower energy, which make them be easily distinguished from their multilayer. The A and B exciton peaks position of SL MoS_2 are about 1.8 eV and 1.95 eV. ReS_2 are direct bandgap semiconductors from monolayer to bulk. The PL spectra of ReS_2 consist of a single narrow feature due to their direct bandgap structure, but the peak position is related to the number of layers. The PL peak position of SL ReS_2 is about 1.6 eV. Our samples in this paper were pre-estimated by the PL measurements.

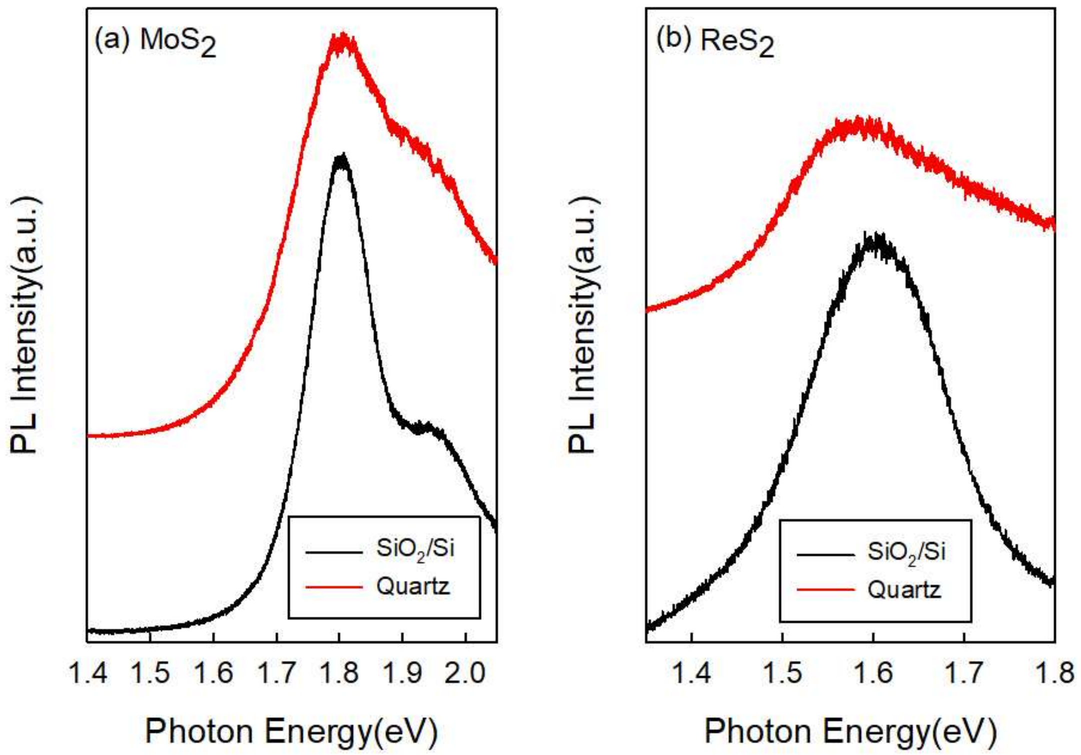

Figure f4. The PL spectra of SL MoS_2 and SL ReS_2 flakes supported on SiO_2/Si substrate and supported on quartz substrate.
